# Diminished gallbladder filling, increased fecal bile acids, and promotion of colon epithelial cell proliferation and neoplasia in fibroblast growth factor 15-deficient mice

**DOI:** 10.18632/oncotarget.25385

**Published:** 2018-05-22

**Authors:** Kunrong Cheng, Melissa Metry, Jessica Felton, Aaron C. Shang, Cinthia B. Drachenberg, Su Xu, Min Zhan, Justin Schumacher, Grace L. Guo, James E. Polli, Jean-Pierre Raufman

**Affiliations:** ^1^ VA Maryland Healthcare System, Baltimore, Maryland, 21201, USA; ^2^ Department of Medicine, Division of Gastroenterology & Hepatology, University of Maryland School of Medicine, Baltimore, Maryland, 21201, USA; ^3^ Department of Pharmaceutical Sciences, University of Maryland School of Pharmacy, Baltimore, Maryland, 21201, USA; ^4^ Department of Surgery, University of Maryland School of Medicine, Baltimore, Maryland, 21201, USA; ^5^ Department of Pathology, University of Maryland School of Medicine, Baltimore, Maryland, 21201, USA; ^6^ Department of Diagnostic Radiology and Nuclear Medicine, University of Maryland School of Medicine, Baltimore, Maryland, 21201, USA; ^7^ Department of Epidemiology & Public Health, University of Maryland School of Medicine, Baltimore, Maryland, 21201, USA; ^8^ Department of Pharmacology and Toxicology, Ernest Mario School of Pharmacy, Rutgers University, Piscataway, New Jersey, 08854, USA; ^9^ Marlene and Stewart Greenebaum Cancer Center, University of Maryland School of Medicine, Baltimore, Maryland, 21201, USA

**Keywords:** colon neoplasia, bile acids, cell proliferation, gallbladder, enterohepatic circulation

## Abstract

Fibroblast growth factor-19 (human FGF19; murine FGF15) suppresses bile acid synthesis. In FGF19 deficiency, diarrhea resulting from bile acid spillage into the colon mimics irritable bowel syndrome. To seek other consequences of FGF19/15 deficiency, we used *Fgf15*^*-/-*^ and wild-type (WT) mice to assess gallbladder filling, the bile acid pool, fecal bile acid levels, and colon neoplasia. We fasted mice for six hours before assessing gallbladder size by magnetic resonance imaging (MRI). We measured bile acid levels in different compartments by enzymatic assay, and induced colon neoplasia with azoxymethane (AOM)/dextran sodium sulfate (DSS) and quantified epithelial Ki67 immunostaining and colon tumors 20 weeks later. *In vivo* MRI confirmed the gross finding of tubular gallbladders in FGF15-deficient compared to WT mice, but fasting gallbladder volumes overlapped. After gavage with a bile acid analogue, *ex vivo* MRI revealed diminished gallbladder filling in FGF15-deficient mice (*P* = 0.0399). In FGF15-deficient mice, the total bile acid pool was expanded 45% (*P* <0.05) and fecal bile acid levels were increased 2.26-fold (*P* <0.001). After AOM/DSS treatment, colons from FGF15-deficient mice had more epithelial cell Ki67 staining and tumors (7.33 ± 1.32 vs. 4.57 ± 0.72 tumors/mouse; *P* = 0.003 compared to WT mice); carcinomas were more common in FGF15-deficient mice (*P* = 0.01). These findings confirm FGF15, the murine homolog of FGF19, plays a key role in modulating gallbladder filling and bile acid homeostasis. In a well-characterized animal model of colon cancer, increased fecal bile acid levels in FGF15-deficient mice promoted epithelial proliferation and advanced neoplasia.

## INTRODUCTION

Active uptake of bile acids from the distal small intestine and feedback inhibition of hepatic bile acid synthesis tightly regulate bile acid homeostasis; normally, each day only a small fraction of intestinal bile acids is excreted in the feces. The ileal apical sodium-dependent bile acid transporter (ASBT; encoded by *SLC10A2*) is a key regulator of bile acid absorption from the distal small intestine into the enterohepatic circulation; active uptake by ASBT and passive absorption throughout the gut results in the recovery of ∼95% of intestinal bile acids. In mouse models, deficiency or impaired function of ASBT increases bile acid spillage into the feces by 6- to 10-fold and strikingly reduces the size of the bile acid pool [1, 2]. ASBT-mediated transport of bile acids into ileal enterocytes also activates a key nuclear bile acid receptor, farnesoid X receptor (FXR), which regulates intestinal synthesis and release of fibroblast growth factor-19 (FGF19 in humans; FGF15 in mice) [3]. FGF19/15 serves as the primary mechanism for feedback inhibition of hepatic bile acid synthesis. In the absence of FGF19/15, hepatic bile acid overproduction may increase intestinal bile acids to a level exceeding ASBT transport (small intestinal uptake) capacity, thereby increasing bile acid spillage into the colon [4].

Increased fecal dihydroxy bile acids, resulting from intestinal bile acid malabsorption (BAM), can cause intermittent or chronic diarrhea. BAM is categorized into three types (BAM Types 1-3) [5]. Type 1 BAM is the result of gross ileal pathology (e.g. surgical resection, Crohn disease). Type 3 BAM may occur after cholecystectomy or vagotomy, or with celiac disease, bacterial overgrowth, or pancreatic insufficiency. Persons with ‘primary’ (Type 2) BAM lack ileal pathology or antecedent or concurrent conditions associated with bile acid spillage into the colon. Primary (Type 2) BAM is an important cause of diarrhea-predominant irritable bowel syndrome (IBS-D), often the most common reason for gastroenterology out-patient visits; estimates suggest up to one-half of persons with IBS-D may have primary BAM [5].

Based on the importance of ASBT for intestinal uptake of bile acids, it seemed logical to consider ASBT deficiency or impairment might be the cause of primary BAM. However, although there are families with germline mutations of *SLC10A2* [6], such mutations are infrequent and do not account for the prevalence of BAM [7]. Instead, studies over the past 5 to 10 years have implicated impaired gut-liver signaling through the axis of FXR–FGF19–FGFR4 (FGF receptor-4 in hepatocytes). Walters and other investigators have obtained convincing data supporting the concept that primary (Type 2) BAM results from reduced feedback inhibition of hepatic bile acid synthesis by diminished release of intestinal FGF19 [8]; insufficient ileal production or release of FGF19 fails to shut off hepatic bile acid synthesis [9]. This failure results in hepatic bile acid overproduction, which is thought to saturate ileal bile acid transporters (primarily ASBT), thereby augmenting spillage of bile acids into the colon [9]. Compared to wild-type (WT) animals, both the bile acid pool and fecal bile acid levels are larger in FGF15-deficient mice, an animal model of human FGF19 deficiency [10].

Epidemiological and animal studies have long associated increased fecal bile acid levels with elevated colon cancer risk [11, 12]. As anticipated from these observations, using a traditional murine colon neoplasia model, compared to wild-type mice, we observed increased aberrant crypt foci and tumor formation in the colons of ASBT-deficient (*Slc10a2*^*-/-*^) mice [13]. To our knowledge, ours was the first report that increased spillage of endogenous bile acids into the colons of otherwise normal mice was sufficient to promote colon neoplasia. Based on these considerations and the estimated prevalence of primary BAM in the general population suggesting millions of Americans are at risk, it seemed important to determine whether FGF19 deficiency also poses a risk for promoting colon neoplasia. In the present study, we used FGF15-deficient mice as an *in vivo* animal model of human FGF19 deficiency and BAM [10]. In these and WT mice, we compared gallbladder resting and filling volumes, the total bile acid pool, and fecal bile acid levels. We used the azoxymethane (AOM)/dextran sodium sulfate (DSS) intestinal neoplasia model to assess the effects of increased fecal bile acids on colon epithelial cell proliferation and neoplasia. As reported herein, we found FGF15-deficient mice were more likely than WT mice to have diminished gallbladder filling, an expanded bile acid pool, increased fecal bile acid levels, augmented colon epithelial cell proliferation, and advanced colon neoplasia.

## RESULTS

**Figure 1:**
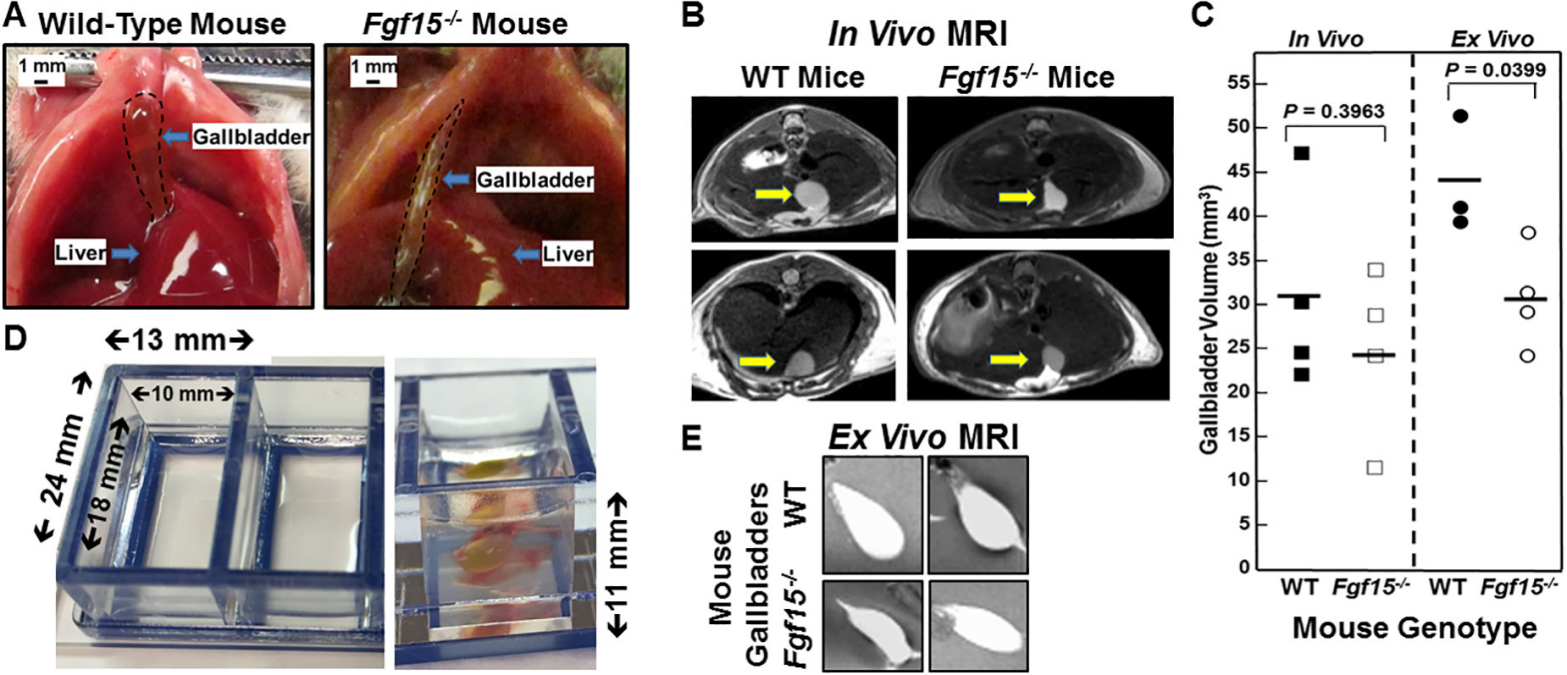
Altered gallbladder shape and diminished gallbladder filling in FGF15-deficient mice **(A)** Photographs of WT and FGF15-deficient mouse gallbladders *in situ* at laparotomy. To improve visual clarity, dashed lines demarcate gallbladders. FGF15-deficient mouse gallbladders were tubular in appearance compared to the spherical gallbladders observed in WT mice. Size bars, 1 mm. **(B)** Representative images from *in vivo* MRI of WT and FGF15-deficient mice show differences in gallbladder shape. Arrows demarcate gallbladders. **(C)** *In vivo* (left) and *ex vivo* (right) measurements of volume of gallbladders from WT and FGF15-deficient mice. Each symbol represents one mouse gallbladder. Horizontal lines demarcate mean values. Mean *ex vivo* volumes were significantly greater in gallbladders from WT compared to FGF15-deficient mice; *P*=0.0399). **(D)** Septate Lucite box used for *ex vivo* imaging of murine gallbladders. Measurements indicate internal and external dimensions of tray containing 2% agarose. Right panel show vertical dimension and gallbladder surrounded by 2% agarose in chamber. **(E)** Representative images from *ex vivo* MRI of gallbladders from WT and FGF15-deficient mouse highlight differences in gallbladder shape.

In addition to modulating hepatic bile acid synthesis, hormonal regulation of gallbladder filling is another putative hormonal function of FGF15 [14]. When examined at laparotomy, Choi et al. reported reduced gallbladder volumes in FGF15-deficient compared to WT mice [14]. As exemplified by the images in Figure 1A, at laparotomy we commonly, but not always, observed differences in gallbladder appearance; gallbladders of fasted FGF15-deficient mice appeared longer and more tubular compared to the fuller, spherical gallbladders of WT mice. Nonetheless, in our experience [15] even minor surgical manipulation of the mouse abdominal cavity may stimulate gallbladder contraction, possibly resulting in underestimation of the organ’s resting volume. To circumvent this potential confounder, we assessed resting gallbladder size and volume in living, fasted FGF15-deficient and WT mice by non-invasive magnetic resonance imaging (MRI).

As shown by representative MRI images in Figure 1B, after a 6-h fast, gallbladders of FGF15-deficient mice tended to have smaller volumes compared to those of WT mice, but there was considerable overlap. When we applied imaging software to calculate gallbladder volumes from the MRI data, mean gallbladder volumes of fasted FGF15-deficient and WT mice were not significantly different (24.22 ± 4.88 vs. 31.08 ± 5.69 mm^3^; mean ± SE for four FGF15-deficient and four WT mice; Figure 1C, left panel).

Next, we assessed the filling capacity of gallbladders from FGF15-deficient and WT mice by gavaging mice with a synthetic bile acid analogue, cholic acid-trifluoroacetyl lysine (CA-lys-TFA), to expand the bile acid pool; we previously described the synthesis, stability, and bioavailability of CA-lys-TFA, and showed that its *in vitro* and *in vivo* transport mimics that of naturally-occurring bile acids [16]. To avoid image obscuration by adjacent abdominal organs and permit side-by-side comparison of gallbladder volumes, we used the novel *ex vivo* approach described in Methods. Interestingly, *ex vivo* gallbladders retained their *in vivo* shape. Representative images of *ex vivo* MRI shown in Figure 1E reveal a more spherical appearance to gallbladders obtained from WT compared to those obtained from FGF15-deficient mice. Moreover, as anticipated following gavage with a large amount of synthetic bile acid, mean *ex vivo* gallbladder volumes were greater than *in vivo* volumes (43.62 ± 3.72 vs. 31.08 ± 5.69 mm^3^ for WT mice and 30.84 ± 2.90 vs. 24.22 ± 4.88 mm^3^ for FGF15-deficient mice) but the differences were not statistically significant. Yet, our *ex vivo* measurements of WT mouse gallbladder volumes by MRI yielded values that were significantly greater than those obtained from FGF15-deficient animals (43.62 ± 3.72 vs. 30.84 ± 2.90 mm^3^; mean ± SE for three WT and four FGF15-deficient mice; *P* = 0.0399; Figure 1C, right panel). We reanalyzed the data shown in Figure 1C as a function of mouse body weight. Mean differences for *in vivo* gallbladder volumes/mouse body weight again failed to achieve statistical significance (*P* = 0.5). However, mean *ex vivo* gallbladder volumes/mouse body weight ratios were significantly lower in FGF15-deficient compared to WT mice (1.158 ± 0.081 vs. 1.549 ± 0.125 mm^3^/g; *P* = 0.0399). Consistent with the previous report [14], our findings using different experimental approaches support the conclusion that FGF15 plays an important role in regulating gallbladder filling.

**Figure 2:**
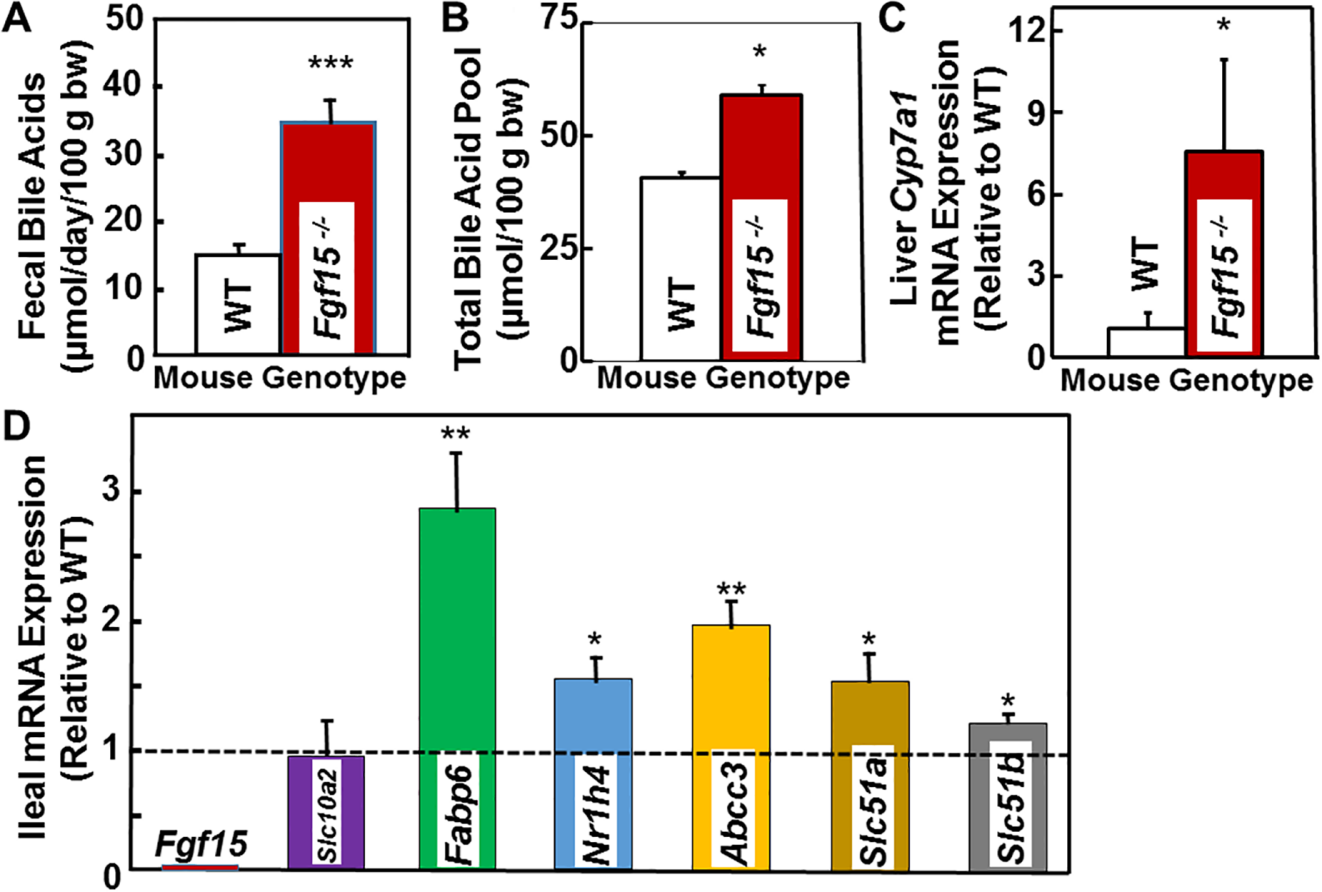
Increased fecal bile acids, total bile acid pool, and expression of hepatic *Cyp7a1* and genes related to bile acid transport in FGF15-deficient mice **(A)** Bar graph shows 2.26-fold increase in fecal bile acids levels in FGF15-deficient compared to WT mice (*P* <0.001). Mean ± SE for five FGF15-deficient and five WT male mice maintained in metabolic cages throughout the three-day stool collection. bw, body weight. **(B)** Bar graph shows 45% increase in the total bile acid pool in FGF15-deficient compared to WT mice (*P* <0.05). **(C)** Bar graph shows changes in hepatic mRNA expression of *Cyp7a1*, an FGF15 target gene. We determined mRNA expression by Q-PCR analysis (in triplicate), normalized using *Gapdh*. We expressed data as mean ± SE *Cyp7a1* mRNA expression in livers from five FGF15-deficient male mice relative to that measured in livers from three WT male mice (set at 1). **(D)** Bar graph shows changes in ileal mRNA expression of genes related to bile acid metabolism [*Nrih4* encodes the farnesoid X receptor (FXR)] and transport [*Slc10a2* encodes ASBT, *Fabp6* encodes intestinal bile acid binding protein (IBABP), *Abcc3* encodes multidrug resistance associated protein-3 (MRP3), and *Slc51a* and *Slc51b* encode organic solute transporters (*Ostα* and *Ostβ*), respectively]. We determined mRNA expression by Q-PCR analysis (in triplicate), normalized using *Gapdh*. We expressed data as mean ± SE ileal mRNA expression for six FGF15-deficient male mice relative to that measured in six WT male mice (set at 1). ^*^P <0.05; ^**^P <0.01; ^***^P < 0.001.

To confirm fecal bile acid concentrations were elevated in FGF15-deficient mice, we placed five WT and five FGF15-deficient male mice in separate metabolic cages to prevent coprophagia, and collected stool for three consecutive days; during this period mice had free access to water and Teklad Global 18% Protein Extruded rodent chow. We measured fecal bile acids using the enzymatic assay described in Methods. As shown in Table 1, for each day of stool collection, bile acid levels were greater in stool collected from FGF15-deficient mice compared to that collected from WT mice. In accord with a previous study [10], we found mean fecal bile acids levels were increased in FGF15-deficient compared to WT mice (34.45 ± 3.52 vs. 15.24 ± 1.00 μmol/day/100 g body weight, respectively; *P* <0.001); combined bile acid levels for the three days were 2.26 ± 0.28-fold greater in FGF15-deficient compared to WT mice (*P* <0.001; Figure 2A). Likewise, we observed a statistically significant, 45% increase in the total bile acid pool of three FGF15-deficient mice compared to three WT mice (59.02 ± 2.38 vs. 40.76 ± 1.00 μmol/100 g body weight, respectively; *P* <0.05; Figure 2B).

**Table 1:** Fecal bile acid outputs for five male WT and five male *Fgf15*^*-/-*^ mice per day over three consecutive days of collection

Day	Fecal Bile Acids (μmol/day/100 g body weight)										KO/WT Ratio
	WT Mice					*Fgf15*^*-/-*^ Mice					
1	13.16	22.08	16.58	13.93	13.40	77.13	37.21	22.59	23.20	31.27	2.26 ± 0.28^***^
2	16.19	12.78	21.55	5.99	15.74	43.13	24.02	24.20	37.48	40.68	
3	15.15	18.71	15.91	12.30	15.16	27.90	25.55	33.87	29.82	38.77	
**Mean ± SE**	15.24 ± 1.00					34.45 ± 3.52^***^					

WT, wild-type; KO, FGF15 knockout mice; ^***^P < 0.001 compared to WT mice.

We used quantitative RT-PCR (Q-PCR) to measure expression of an FGF15 target gene (*Cyp7a1*) in the livers of three WT and five FGF15-deficient mice, and genes involved in intestinal bile acid transport in the ileums of six WT and six FGF15-deficient male mice. We observed a seven-fold increase in hepatic *Cyp7a1* expression in FGF15-deficient mice, compatible with the lack of feedback repression of that gene (Figure 2C). As expected, in FGF15-deficient mice we did not detect ileal mRNA expression of *Fgf15* (Figure 2D). Moreover, as shown in Figure 2D, in ileums from FGF15-deficient compared to WT mice we detected no difference in ileal mRNA expression of *Slc10a2*, encoding ASBT. In contrast, in ileums from FGF15-deficient mice, we detected significant increases in mRNA expression of genes encoding the farnesoid X receptor (FXR encoded by *Nrih4*). Likewise, we detected increased expression of transporters that help move bile acids from the cytoplasm of ileal enterocytes across the basolateral membrane to the enterohepatic circulation; intestinal bile acid binding protein (IBABP encoded by *Fabp6*), multidrug resistance-associated protein-3 (MRP3 encoded by *Abcc3*), and organic solute transporters (OSTα and OSTβ, encoded by *Slc51a and Slc51b*, respectively) These findings are similar to those published by others who examined FGF15-deficient mice [10]. Significant increases in expression of FXR and FXR-regulated genes in the ileum such as those for IBABP, MRP3, and OSTα/β is consistent with an expanded bile acid pool and increased bile acid levels in enterocytes of FGF15-deficient mice. Moreover, these findings are compatible with recent findings suggesting increased ileal bile acid transporter expression helps protect ileal enterocytes from accumulating potentially toxic bile acid levels [2].

**Figure 3:**
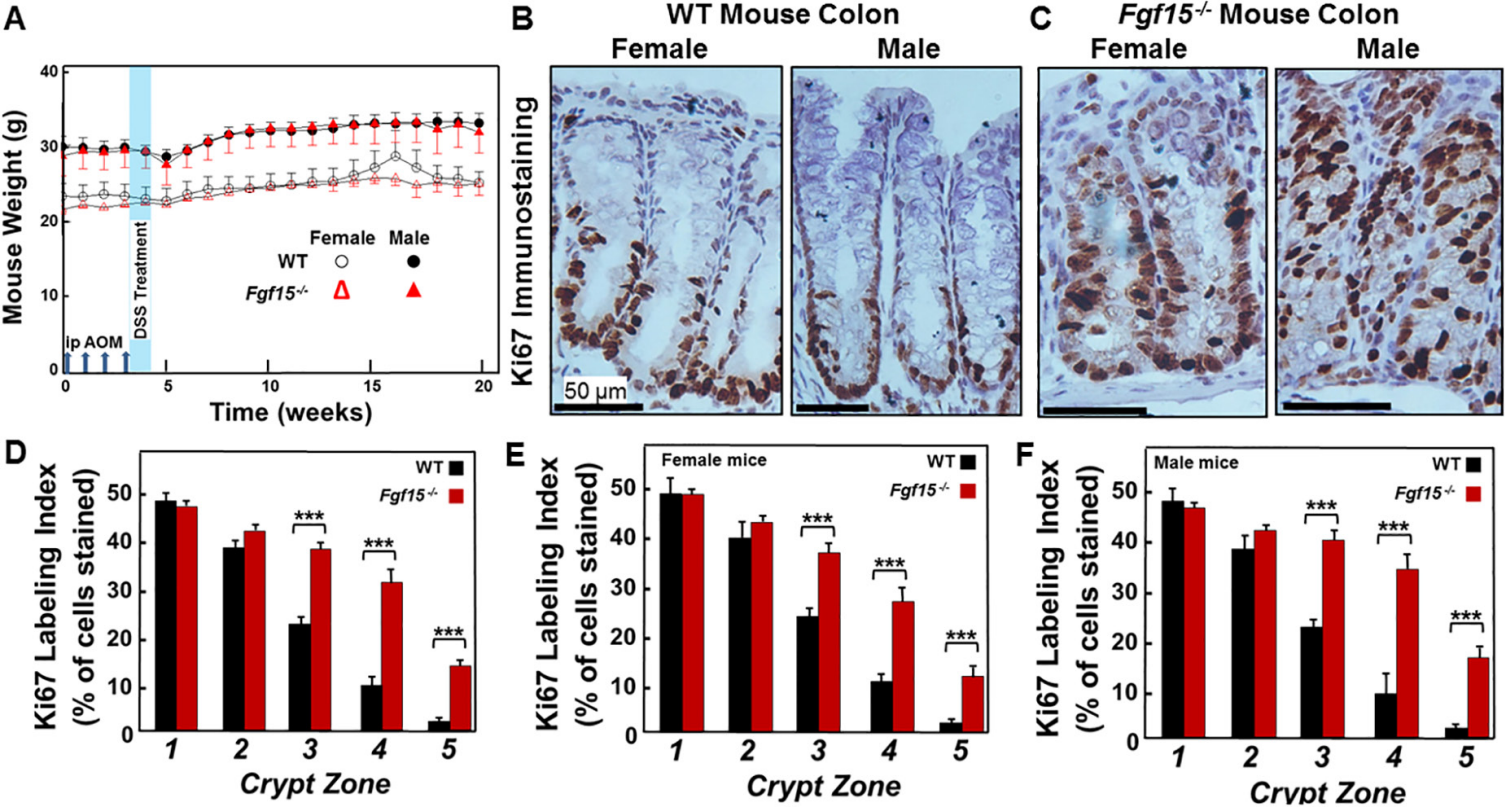
Experimental design and effects of FGF15 deficiency on colon epithelial cell proliferation in AOM/DSS-treated mice **(A)** Experimental design and mouse weights over the course of the 20-week study. We weighed mice weekly. Vertical arrows on the horizontal axis indicate intraperitoneal injection with 7.5 mg/kg AOM/kg body weight at day 1, 8, 15, and 22. Vertical blue band indicates that we added 2.5% dextran sulfate sodium (DSS) to the drinking water of AOM-treated mice on days 29 through 33. Fourteen WT and 15 *Fgf15*^*-/-*^ mice were treated with AOM plus DSS. Error bars denote SE. There were no differences between the weights of WT and FGF15-deficient mice. However, regardless of genotype, male mice weighed 10-20% more than female mice. **(B** and **C)** Representative images of Ki67 immunostaining in crypts from female and male WT (B) and *Fgf15*^*-/-*^ (C) mouse colons. Note increased Ki67 immunostaining in upper portions of crypts from FGF15-deficient mice. **(D-F)** FGF15 deficiency is associated with increased colon epithelial cell proliferation. An investigator masked to genotype measured the Ki67 labeling index in normal crypts from the distal half of each mouse colon. Crypts were divided into five equal zones, starting from the proliferative area at the base (zone 1) and extending to the luminal surface (zone 5), and immunostaining was assessed in approximately 1000 colon epithelial cells from each mouse. (D) Results for 14 WT and 15 *Fgf15*^*-/-*^ mice; (E) Results for 6 WT and 7 *Fgf15*^*-/-*^ female mice; (F) Results for 8 WT and 8 *Fgf15*^*-/-*^ male mice. We observed significant increases in Ki67 staining in the upper portions of crypts in non-neoplastic segments of colon from FGF15-deficient compared to WT mice overall and in female and male mice evaluated separately. (^***^P < 0.001).

As shown in Figure 3A, which schematizes our experimental approach to inducing colon neoplasia, the weights of age-matched WT and FGF15-deficient mice of the same gender were similar. Regardless of genotype, female mice weighed 10-20% less than males, a difference that persisted throughout the study period including a transient drop in body weight immediately after DSS treatment. We maintained 8 female and 4 male WT, and 8 female and 9 male FGF15-deficient untreated mice alive for more than one year – none of the 12 WT and 17 FGF15-deficient mice not treated with carcinogens developed colon tumors (Table 2). Whereas all AOM/DSS-treated WT mice developed colon tumors, two WT mice treated with only 7.5 mg AOM/kg body weight (no DSS) had not developed colon tumors when euthanized 20 weeks after the first AOM injection, confirming combined treatment with AOM and DSS is required to induce colon neoplasia consistently.

**Table 2:** Summary of animal studies of colon epithelial cell proliferation and neoplasia

Study	Genotype	Sex	N	Key Experimental Outcomes
Epithelial Cell Proliferation	WT	Both	14	In *Fgf15*^*-/-*^ mice, we detected a shift of proliferating cells to upper crypt zones (*P* <0.001).
		M	8	
		F	6	
	*Fgf15*^*-/-*^	Both	15	
		M	8	
		F	7	
No Carcinogen Treatment	WT	Both	12	After >1 year surveillance without carcinogen treatment, no WT or *Fgf15*^*-/-*^ mouse developed colon neoplasia.
		M	4	
		F	8	
	*Fgf15*^*-/-*^	Both	17	
		M	9	
		F	8	
Colon Tumor Induction (AOM/DSS)	WT	Both	14	Colon tumors were detected in 27 of 29 mice; *Fgf15*^*-/-*^ mice had more colon tumors overall (*P* = 0.003), and in males (*P* = 0.03) and females (*P* < 0.05) considered separately.
		M	6	
		F	8	
	*Fgf15*^*-/-*^	Both	15	
		M	7	
		F	8	

WT, wild-type; N, number of mice; M, male; F, female.

Increased cell proliferation is a prerequisite for neoplastic transformation [17]; in both *in vivo* and *in vitro* experiments, increased levels of bile acids stimulate colon epithelial cell proliferation [13, 18]. We used Ki67 immunostaining to assess the impact of FGF15 deficiency and the consequent increase in fecal bile acids on colon epithelial cell proliferation. As shown in Figure 3B-3F, FGF15 deficiency differentially affected Ki67 immunostaining depending upon the crypt zone examined. In colons from *Fgf15*^*-/-*^ compared to those from WT mice, there were similar numbers of Ki67-stained cells at the crypt base (zones 1 and 2); however, cells closer to the intestinal lumen (zones 3-5) demonstrated significantly more Ki67 staining in FGF15-deficient compared to WT mice (*P* <0.001; Figure 3D). This difference was most striking in crypt zone 5, the most proximate to the lumen, where there was a greater than six-fold increase in Ki67 immunostained colon crypt cells in tissues from FGF15-deficient compared to those from WT mice (Figure 3D; 13.6 ± 1.8 vs. 2.2 ± 0.9% of cells stained; *P* <0.001). As in ASBT deficiency [13], we believe the shift of proliferating cells to the upper crypt zones in *Fgf15*^*-/-*^ mice represents an early manifestation of hyperproliferation predisposing cells to dysplasia (Table 2).

In an attempt to mitigate the common failure of clinical trials to validate animal research findings, the National Institutes of Health developed policies that require inclusion of male and female animals in preclinical studies [19]. To comply with this requirement, we performed subgroup analyses of female and male FGF15-deficient and WT mice (Figure 3E/3F). The findings of this subgroup analysis mirrored those for combined mouse genders (Figure 3D); in both male and female *Fgf15*^*-/-*^ mice we observed a shift of proliferating cells to upper crypt zones (Table 2). Collectively, these findings support the role of fecal bile acids as growth factors that promote colon epithelial cell proliferation.

**Figure 4:**
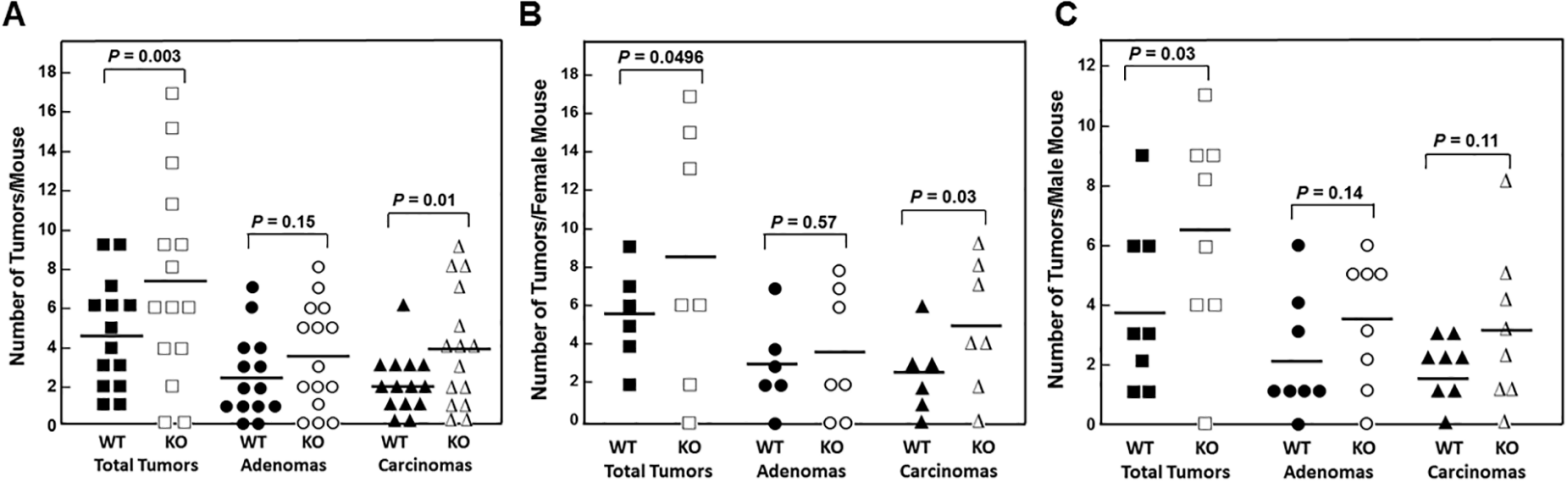
FGF15 deficiency promotes colon neoplasia **(A)** Numbers of overall tumors, adenomas, and adenocarcinomas in the colons of 14 WT and 15 *Fgf15*^*-/-*^ mice treated with AOM plus DSS. Each symbol represents the number of tumors, adenomas, and adenocarcinomas (carcinomas) in one mouse colon; horizontal bars indicate means. WT and *Fgf15*^*-/-*^ mouse colons contained a mean of 4.57 and 7.33 tumors, 2.50 and 3.47 adenomas, and 2.07 and 3.87 adenocarcinomas, respectively (*P* = 0.003, 0.15, and 0.01). **(B)** Numbers of overall tumors, adenomas, and adenocarcinomas in the colons of 6 WT and 7 *Fgf15*^*-/-*^ female mice treated with AOM plus DSS. Each symbol represents number of tumors, adenomas, and carcinomas in one female mouse; horizontal bars indicate means. WT and *Fgf15*^*-/-*^ female mouse colons contained a mean of 5.50 and 8.43 tumors, 3.00 and 3.57 adenomas, and 2.50 and 4.86 adenocarcinomas, respectively (*P* = 0.0496, 0.57, and 0.03). **(C)** Numbers of overall tumors, adenomas, and carcinomas in the colons of 8 WT and 8 *Fgf15*^*-/-*^ male mice treated with AOM plus DSS Each symbol represents number of tumors, adenomas, and adenocarcinomas in one male mouse; horizontal bars indicate means. WT and *Fgf15*^*-/-*^ male mouse colons contained a mean of 3.88 and 6.38 tumors, 2.13 and 3.38 adenomas, and 1.75 and 3.00 adenocarcinomas, respectively (*P* = 0.03, 0.14, and 0.11).

All WT and all but two FGF15-deficient animals developed colon tumors (adenomas and adenocarcinomas; Figure 4A and Table 2). FGF15 deficiency was associated with a 60% increase in the mean number of colon tumors per mouse in FGF15-deficient compared to WT mice (7.33 ± 1.32 vs. 4.57 ± 0.72 colon tumors per mouse, respectively; *P* = 0.003). Grossly, we differentiated adenomas from adenocarcinomas by their smaller size (generally less than 3.5 mm in largest diameter), smooth contour, and pale tan rather than red color. When we assessed the distribution of tumor pathology, we found the mean number of adenomas was increased in FGF15-deficient compared to WT mice (3.47 ± 0.70 vs. 2.50 ± 0.57 colon adenomas per mouse, respectively), a difference that did not achieve statistical significance (*P* = 0.15). However, the mean number of adenocarcinomas was significantly greater in FGF15-deficient compared to WT mice (3.87 ± 0.77 vs. 2.07 ± 0.41 colon carcinomas per mouse, respectively; *P* = 0.01). These findings were consistent with our report that AOM/DSS-treated ASBT-deficient (*Slc10a2*^*-/-*^) mice also demonstrated a significant increase in colon adenocarcinomas but not adenomas [13].

As shown in Figure 4B and 4C, findings in female and male mice individually were similar to the combined data; FGF15 deficiency was associated with a 53% increase in the mean number of colon tumors in female FGF15-deficient compared to WT mice (8.43 ± 2.50 vs. 5.50 ± 0.99 colon tumors per mouse, respectively; *P* = 0.0496). Likewise, FGF15 deficiency was associated with a 64% increase in the mean number of colon tumors in male FGF15-deficient compared to WT mice (6.38 ± 1.27 vs. 3.88 ± 1.01 colon tumors per mouse, respectively; *P* = 0.03). Mean numbers of adenomas and carcinomas were increased in both female and male FGF15-deficient mice (Figure 4B and 4C; Table 2). Most likely because subgroup numbers were small, except for carcinomas in female mice (4.86 ± 1.24 and 2.50 ± 0.85 carcinomas per FGF15-deficient and WT mice, respectively; P = 0.03), these differences did not achieve statistical significance at the 0.05 level.

**Figure 5:**
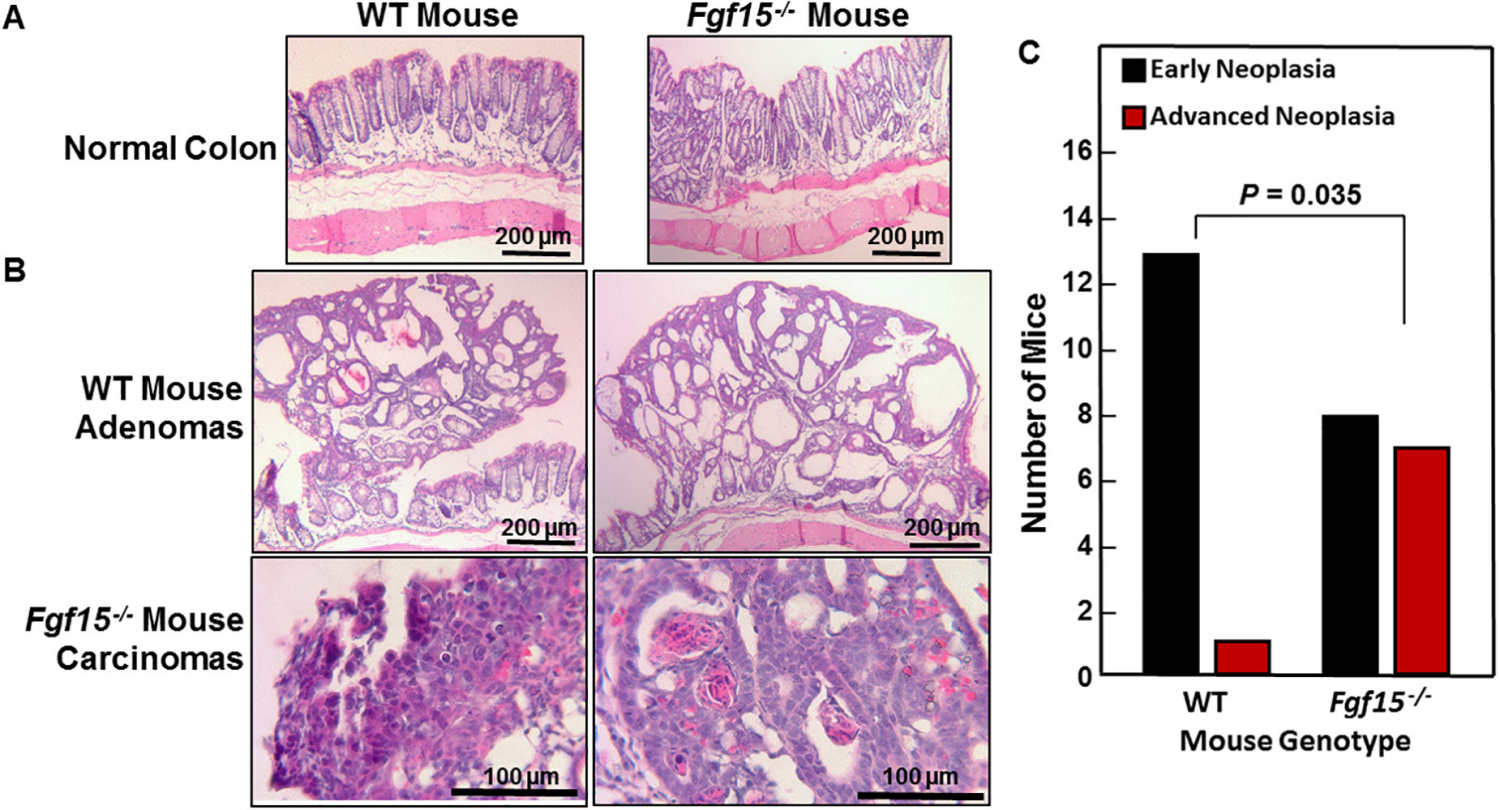
Advanced colon neoplasia in FGF15-deficient mice **(A)** Representative images of normal colons from WT and FGF15-deficient mice. Size bars, 200 μm. **(B)** Representative images of colon adenomas in two WT mice and adenocarcinomas in two FGF15-deficient mice. Size bars, 200 and 100 μm, respectively. **(C)** Numbers of WT and FGF15-deficient mice whose colons contained only early neoplasia [aberrant crypt foci (ACF) and/or adenomas] versus those containing advanced neoplasia (high-grade dysplasia and/or adenocarcinoma). The distribution of these lesions in WT versus FGF15-deficient mice was significantly different (*P* = 0.035, two-tailed Fisher’s exact test).

As illustrated by representative sections shown in Figure 5A, we observed no morphological differences between non-neoplastic sections of WT and FGF15-deficient mouse colons. Figure 5B shows representative adenomas and adenocarcinomas from WT and FGF15-deficient mice, respectively. For analytical purposes, we segregated colon neoplasia into two categories, early neoplasia, consisting of aberrant crypt foci (ACF) and adenomas, versus advanced neoplasia, consisting of high-grade dysplasia and adenocarcinomas [20]. As shown in Figure 5C, our senior pathologist detected advanced neoplasia in sections from only one of 14 WT mice. In contrast, ACF/adenomas and advanced pathology were distributed almost equally in sections from FGF15-deficient mice (eight mice with early neoplasia versus seven with advanced neoplasia; *P* = 0.035 compared to the distribution of tumor pathology in WT mice, two-tailed Fisher’s exact test) (Figure 5C). These histological findings confirm the data shown in Figure 4A from gross inspection of colons immediately after euthanasia. In addition to having more colon tumors, FGF15-deficient mice were more likely to have tumors with advanced neoplasia.

Muscarinic receptors and ligands, including bile acids, play important roles in colon neoplasia, and human colon adenomas and adenocarcinomas over-express M3 muscarinic receptors (M3R) [21]. In animal models, M3R knockout or inactivation attenuates murine colon epithelial cell proliferation and neoplasia [22, 23]. Our previous work suggests bile acids stimulate colon cancer cell proliferation and survival by functional interaction with M3R [18, 24–26]. Since our previous study of WT and ASBT-deficient mice showed increased M3R expression in tumors from both genotypes [13], we examined M3 muscarinic receptor (M3R) expression using immuno-histochemistry in sections from normal colon epithelium and tumors from WT and FGF15-deficient mice. In both WT and FGF15-deficient mice, M3R staining was significantly increased in colon tumors compared to normal colon epithelium (2.66 ± 0.12 and 1.89 ± 0.08 arbitrary units for tumors and normal epithelium, respectively, from WT mice; 2.94 ± 0.11 and 2.03 ± 0.11 arbitrary units for tumors and normal epithelium, respectively, from FGF15-deficient mice; *P* <0.001 for both comparisons). Although M3R expression in tumors from FGF15-deficient mice was approximately 10% greater than that in tumors from WT mice, this difference did not achieve statistical significance (*P*=0.0887). With regard to these differences in M3R expression, we had previously found that activating M3R signaling with bethanechol in AOM-treated male WT mice increased tumor number by 30% [27], similar to what we observed in FGF15-deficient mice (Figure 4C). As observed with FGF15 deficiency (Figure 4A and 5C), M3R activation with bethanechol increased the number of colon adenocarcinomas, not adenomas [27].

## DISCUSSION

FGF15-deficient mice represent a useful preclinical model to study human FGF19 deficiency, the most common cause of primary bile acid malabsorption [10]. Deficiency of either FGF19 or FGF15, intestinal hormones that serve as feedback brakes on hepatic bile acid production, results in increased bile acid synthesis and enlargement of the bile acid pool. FGF19-deficient humans and FGF15-deficient mice have expanded bile acid pools and elevated levels of fecal bile acids (Figure 2A and 2B; Table 1) [10].

In the current study, we used innovative *in vivo* and *ex vivo* imaging-based approaches to extend the previous observation that FGF15 regulates gallbladder function [14]. Our findings confirmed FGF15 deficiency alters gallbladder shape and filling (Figure 1); in response to an exogenous bile acid load, FGF15-deficient mice had significantly reduced gallbladder filling (Figure 1C). Conceptually, both increased hepatic synthesis of bile acids and diminished gallbladder filling are likely to contribute to the increased load of bile acids that reaches the ileum, overwhelms the transport capacity of ASBT, and spills into the colon. We confirmed FGF15-deficient mice have both an enlarged bile acid pool and increased levels of fecal bile acids. However, the fold-increase in fecal bile acids associated with FGF15 deficiency (2.26-fold) was less than that observed in ASBT-deficient mice (6- to 10-fold) [2, 13].

We used FGF15-deficient mice to determine if more modest increases in fecal bile acid levels than those observed in ASBT-deficient mice were sufficient to stimulate colon epithelial cell proliferation and neoplasia. Surprisingly, we found that even a modest 2.26-fold increase in fecal bile acids was sufficient to increase colon epithelial cell proliferation. Ki67 immunostaining, an index of cell proliferation, was increased significantly in colon epithelium from FGF15-deficient mice (Figure 3). Notably, there were 60% more colon tumors in FGF15-deficient mice, and advanced neoplasia was significantly more prevalent (Figures 4 and 5).

Our findings may have important clinical implications. First, conditions associated with diminished gallbladder filling, as observed in FGF15-deficient mice, are associated with an increase in gallbladder disease, primarily the formation of gallstones and resultant diseases, including cholecystitis, choledocholithiasis, and acute pancreatitis, amongst others. Pregnancy, a common condition associated with diminished gallbladder filling [28], is associated with an increased propensity to gallstone formation, gallbladder disease, and pancreatitis [29]. The risk of cholelithiasis is also increased in Crohn disease affecting the terminal ileum and following ileal resection [30]; in these conditions, FGF19 deficiency as a consequence of intestinal mucosal inflammation or absence is likely to impair gallbladder function. In the long-term, chronic cholelithiasis may increase the risk of developing gallbladder cancer, likely due to persistent low-grade inflammation [31]. Our findings suggest that individuals with FGF19 deficiency may be at increased risk of gallstone formation, a speculation that requires confirmation by clinical studies.

The present report also represents an important extension of our previous finding that increased fecal bile acid levels resulting from ASBT deficiency promotes colon epithelial proliferation and neoplasia [13]. We now show that even lower levels of bile acid spillage into the colon pose a risk for colon neoplasia – whereas ASBT deficiency is associated with a 6- to 10-fold increased fecal bile acids [1, 2], FGF15 deficiency causes a more modest 2- to 3-fold increase in fecal bile acid levels ([10]; Figure 2A; Table 1). In a commonly used animal model of sporadic colon neoplasia, we show FGF15 deficiency is associated with a significant increase in colon neoplasia; in both male and female FGF15-deficient compared to WT mice, the overall number of colon tumors was increased significantly (Figure 4). Moreover, in line with our previous observation that fecal bile acids are tumor promoters, not initiators [13], we observed significant increases in the numbers of carcinomas but not adenomas (Figures 4 and 5).

Whereas we acknowledge the limitations of extrapolating murine data to human disease [32], our finding that modest increases in the spillage of *endogenous* bile acids into the colon can promote neoplasia raises additional clinical concerns [13]. As noted in the Introduction, FGF19 deficiency appears to be a much more common cause of bile acid malabsorption in humans than deficient or mutated ASBT [7–9]; primary bile acid malabsorption caused by FGF19 deficiency may account for up to one-half of persons with IBS-D [5]. Based on current estimates this would represent more than 20 million people in the United States [5]. Bile acid malabsorption is also common in inflammatory bowel diseases (primarily Crohn disease involving the terminal ileum), after bariatric surgery (primarily Roux-en-Y gastric bypass), and as a side effect of some drugs approved by the U.S. Food and Drug Administration (e.g. lenalidomide); these drugs inhibit the function of ASBT, the key intestinal bile acid transporter [13, 33].

Spillage of dihydroxy bile acids into the colon stimulates defecation and, to maintain bile acid pool homeostasis, increased fecal loss of bile acids stimulates their *de novo* synthesis in the liver. As bile acids are by-products of cholesterol metabolism, increased hepatic synthesis of bile acid reduces serum cholesterol levels. Hence, the pharmaceutical industry is targeting intestinal bile acid transport to treat common medical conditions; constipation, hypercholesterolemia, and nonalcoholic fatty liver disease ([34]; reviewed in [35]). Thus, our previous [13] and current findings raise the possibility that therapeutic approaches targeting bile acid transport may have the unanticipated side effect of promoting colon neoplasia.

Finally, emerging data from clinical correlations and *in vitro* experiments suggest that hormonal signaling by intestinal FGF19 through its hepatic receptor, FGFR4, plays a role in promoting liver carcinogenesis [36, 37]. Consequently, the pharmaceutical industry is targeting the FGF19 signaling pathway to treat hepatocellular carcinoma (see ClinicalTrials.Gov identifiers NCT00825955 and NCT01215739) [38]. Selective or non-selective inhibitors of FGF19 and/or FGFR4 have the potential to increase fecal bile acid levels and, based on our findings, promote colon neoplasia. Given the current limited life expectancy for patients with liver cancer, this may not be an important concern, but if treatment responses become more durable, off-target effects on the colon may require consideration.

The central conclusion of our current and previous work is that persons with increased fecal bile acid levels may be at risk for more rapid progression of colon neoplasia [13]. Whether these conditions warrant more frequent and earlier initiation of colon cancer screening remains to be determined. Admittedly, such concerns remain speculative until informed by additional epidemiological or clinical studies. Nonetheless, we believe our results are sufficiently concerning to consider additional clinical studies and post-marketing surveillance for colon adenomas and adenocarcinomas to assess the risks of drugs whose mechanisms of action will purposefully or inadvertently increase the spillage of bile acids into the colon.

## MATERIALS AND METHODS

### Animals

FGF15 knockout (KO, *Fgf15*^*-/-*^) mice on a pure C57BL/6J genetic background do not survive embryogenesis but are viable and fertile on a mixed genetic background (25% C57BL/6; 75% 129SvJ) [39]. For these studies, we used both male and female wild-type (WT) and FGF15 knockout (KO) mice backcrossed on this mixed genetic background. We housed mice under identical conditions in a pathogen-free environment with a 12:12-hour light/dark cycle and free access to water and standard mouse chow (Teklad Global 18% Protein Extruded Rodent Diet contains 24% protein, 18% fat, and 58% fiber as a percentage of calories). All animal studies were conducted in accordance with the *Guide for the Care and Use of Laboratory Animals* prepared by the U.S. National Academy of Sciences and approved by both the Institutional Animal Care and Use Committee at the University of Maryland School of Medicine and the Research and Development Committee at the VA Maryland Health Care System.

### Live-animal magnetic resonance imaging (MRI)

After fasting for six hours, mice were anesthetized with ketamine/xylazine administered via an intraperitoneal (IP) catheter placed before imaging; maintenance doses of ketamine/xylazine were infused approximately every 20 min during imaging [40]. All *in vivo* and *ex vivo* MRI experiments were performed using a Bruker BioSpec 70/30USR Avance III 7T horizontal bore MR scanner (Bruker Biospin MRI GmbH, Germany), equipped with a BGA12Sgradient system and interfaced to Bruker Paravision 5.1 for image acquisition and processing. We used a Bruker 40-mm volume radio frequency coil for data acquisition. We placed the anesthetized mice prone in a Bruker animal bed, centered the imaging coil over the liver to cover the gallbladder, and moved the animal bed to the center of the magnet. We used an MR-compatible small-animal monitoring system to monitor the mouse’s respiration rate and body temperature. We maintained body temperature at 36 to 37.5°C using warm water circulation. We acquired ^1^H MR images using RARE (rapid acquisition with relaxation enhancement) sequence in the cross view of the sample or the body of the animal with repetition time 1850 ms, echo time 25.74 ms, RARE factor 8, field of view 40 × 40 mm^2^, slice thickness 0.5 mm, matrix size 200 x 200, in-plane resolution 200 × 200 μm^2^, number of slices 20, and number of averages 24.

### *Ex vivo* gallbladder MRI and volume measurements

Mice were gavaged with a synthetic bile acid, cholic acid-trifluoroacetyl lysine (CA-lys-TFA, 150 mg/kg) [41]. Eight and one-half hours after gavage and 6 h after fasting, we anesthetized mice with ketamine/xylazine administered IP. Gallbladders were removed by a method described in a video offered on-line by the Journal of Visualized Experiments [15], and placed on a layer of 2% agarose in a Lab-Tek II chamber (Thermo Scientific). We covered excised gallbladders with additional 2% agarose and refrigerated the chambers at 4°C until we performed MRI. Within 18 h of gallbladder resection, we imaged gallbladders *ex vivo* using the MRI protocol described above. After imaging, we transferred all *in vivo* and *ex vivo* imaging data to an off-line server where we performed image analysis. The high signal regions of the gallbladder were manually drawn in the Medical Image Processing, Analysis, and Visualization software (MIPAV v5.3.1, CIT, NIH, Bethesda, MD) [42]. We then calculated gallbladder volume (mm^3^) using MIPAV software.

### Quantitative real-time polymerase chain reaction

To examine changes in the expression of genes relevant to bile acid transport, we extracted total RNA from segments of terminal ileum stored in RNA*later*. We used 5 μg RNA for first-strand cDNA synthesis using the Superscript III First Strand Synthesis System for RT-PCR (Invitrogen). We performed quantitative real-time PCR (Q-PCR) with 50 ng cDNA, the SYBR Green PCR Master Mix (Applied Biosystems) and specific primers at a final concentration of 0.5 μM in 20 μl sample volumes. We used primer sequences validated in previous studies of bile acid transport (Table 3) [2, 39, 43]. We performed Q-PCR using the 7900HT Fast System and SYBR Green Master Mix (ABI). PCR conditions were 5 min at 95°C followed by 37 cycles of 95°C for 15 sec, 60°C for 20 sec, and 72°C for 40 sec and a final cycle at 95°C for 15 sec, 60°C for 15 sec, and 95°C for 15 sec. We analyzed data using ABI instrument software (SDS 2.1) and used *Gapdh* expression to normalize results. We ran all samples in triplicate and used the *C*_*T*_ (2^–ΔΔCT^) method to compare relative transcript expression.

**Table 3:** PCR primers used in this study

Protein	Gene	Forward primer	Reverse primer
FGF15	*Fgf15*	5' GCCATCAAGGACGTCAGCA 3'	5' CTTCCTCCGAGTAGCGAATCAG 3'
ASBT	*Slc10a2*	5’ TGGGTTTCTTCCTGGCTAGACT 3’	5’ TGTTCTGCATTCCAGTTTCCAA 3’
IBABP	*Fabp6*	5’ CAAGGCTACCGTGAAGATGGA 3’	5’ CCCACGACCTCCGAAGTCT 3'
FXR	*Nr1h4*	5' TCCACAACCAAGTTTTGCAG 3'	5' TCTCTGTTTGTTGTACGAATCCA 3'
MRP3	*Abcc3*	5' AGAGCTGGGCTCCAAGTTCT 3'	5' TGGTGTCTCAGGTAAAACAGGTAGCA 3'
OSTα	*Slc51a*	5' TACAAGAACACCCTTTGCCC 3'	5' CGAGGAATCCAGAGACCAAA 3'
OSTβ	*Slc51b*	5' GTATTTTCGTGCAGAAGATGCG 3'	5' TTTCTGTTTGCCAGGATGCTC 3'
CYP7A1	*Cyp7a1*	5' AACAACCTGCCAGTACTAGATAGC 3'	5' GTGTAGAGTGAAGTCCTCCTTAGC 3'

### Fecal bile acids and bile acid pool measurements

For measurement of fecal bile acids, the apparent concentration of 3α-hydroxy bile acids was determined enzymatically using the method described by Porter et al. for fecal bile acids [44]. Briefly, we refluxed lyophilized stool (100 mg) for 120 min in ethylene glycol-0.7 M potassium hydroxide for bile acid desorption as well as deconjugation. After cooling, we acidified the solution, and extracted bile acids with diethyl ether. The ether extract was evaporated, the residue re-dissolved in methanol, and its bile acid content determined by the enzymatic procedure. The alkaline hydrolysis-ether extraction procedure used in this method gives complete recovery of fecal bile acids [44, 45]. The experimental design involved daily fecal collection for three consecutive days from five mice per genotype maintained in metabolic cages for the duration of daily stool collection.

Total bile acid pool size was determined by analyzing gallbladders, small intestines, and livers harvested from WT and FGF15-deficient mice. We homogenized gallbladders in 1 ml of PBS, diluted homogenates 10x in PBS, and determined bile acid concentration using the Total Bile Acid Assay kit (Diazyme, Poway, CA) according to the manufacturer’s protocol. We weighed and homogenized livers in 0.5 ml of water, added 0.5 ml of 100% ethanol to each homogenate, rotated the samples for one hour before centrifugation, collected supernatants, and resuspended pellets in 1 ml of 100% ethanol for repeat rotation and centrifugation. We combined supernatants and measured bile acid concentration using the Total Bile Acid Assay kit; we normalized the concentration of bile acids to the weights of the initial liver homogenates. Likewise, we homogenized the entire small intestine in 3 ml of water and added 2 ml of 100% ethanol; these samples were then treated and bile acids measured and normalized as described for the liver. Finally, we normalized total bile acid pool levels to mouse body weight.

### Colon neoplasia study

*Fgf15*^*-/-*^ mice (N=15) and WT mice (N=14) were treated with 7.5 mg/kg body weight azoxymethane (AOM) by intraperitoneal injection weekly for four weeks beginning at 10 to 23 weeks of age (times of AOM initiation were matched between *Fgf15*^*-/-*^ and WT mice). After the last AOM injection, we supplemented the animals’ drinking water with 2.5% dextran sulfate sodium (DSS) for five days. We confirmed the need for DSS in a pilot experiment using two male WT mice treated with 7.5 mg/kg body weight AOM alone (no DSS); neither mouse developed colon neoplasia. Mice were euthanized 20 weeks after the first AOM injection; after harvest, we measured colon length, and opened the colons longitudinally for gross visual inspection by an investigator masked to genotype and treatment who measured tumor number and size using digital calipers. We characterized tumors as adenomas or adenocarcinomas based on size, contour, and color, as validated previously [13]. We photographed colon segments using a Nikon SMZ1500 dissecting microscope. Tumor volume was calculated using the equation, volume = ½ (length X width^2^) [23]. Sections of normal colon and tumors were fixed in 4% paraformaldehyde and paraffin-embedded; five-micron sections were used for hematoxylin and eosin (H&E)- and immuno-staining. A senior pathologist masked to mouse genotype and treatment classified colon tumors in these H&E-stained sections as adenomas or adenocarcinomas according to consensus recommendations [20].

### Ki67 immunohistochemical staining

To examine cell proliferation, Ki67 staining was performed as described by Holt et al. [46] Only the distal half of the colon and complete crypts were analyzed by investigators masked to genotype and treatment. To measure changes in the distribution of Ki67 staining, we divided crypts into five equal zones, starting at the base (zone 1) and extending to the luminal surface (zone 5). The ‘labeling index’ was calculated as the percentage of cells in a crypt zone that stained for Ki67 [46]; approximately 1,000 colon epithelial cells were evaluated per mouse colon.

### M3 subtype muscarinic receptor (M3R) staining

To identify and quantify protein expression, we immunostained formalin-fixed paraffin-embedded tissue sections with specific antibodies against M3 muscarinic receptors (Alomone Labs, Jerusalem, Israel) [13]. We examined tumor sections with a Nikon 80i photomicroscope at 200X magnification. A senior pathologist, masked to tissue origin, reviewed and scored sections. To minimize variation, we examined and photographed all tissue sections using the same microscope settings.

### Statistical analysis

We present data as average values ± standard error (SE). We used the unpaired Student's *t* test (assuming unequal variance) to compare continuous variables between two independent groups. For multi-group comparisons, we applied two-way ANOVA with one between-subject factor (WT versus FGF15-deficient) and one within-subject factor (normal tissue versus tumor tissue) followed by *post hoc* tests with Tukey-Kramer’s adjustment for *P* values. We used Fisher’s exact test to compare proportions. We considered differences significant when *P* was less than 0.05.

## Abbreviations

ACF: aberrant crypt foci
AOM: azoxymethane
ASBT: apical sodium-dependent bile acid transporter
BAM: bile acid malabsorption
CA-lys-TFA: cholic acid-trifluoroacetyl lysine
DSS: dextran sodium sulfate
FGF: fibroblast growth factor
FGFR4: (FGF receptor-4)
FXR: farnesoid X receptor
H&E: hematoxylin and eosin
KO: knockout
M3R: type 3 muscarinic receptors
MRI: magnetic resonance imaging
Q-PCR: quantitative real-time polymerase chain reaction
SE: standard error
WT: wild-type
